# 癌相关成纤维细胞表型转换的遗传学与表观遗传学机制研究现状

**DOI:** 10.3779/j.issn.1009-3419.2015.02.12

**Published:** 2015-02-20

**Authors:** 大理 陈, 国卫 车

**Affiliations:** 610041 成都，四川大学华西医院胸外科 Department of Thoracic Surgery, West China Hospital, Sichuan University, Chengdu 610041, China

**Keywords:** 癌相关成纤维细胞, 表观遗传学, 表型转换型, Cancer-associated fibroblasts, Epigenetics, Phenotype transformation

## Abstract

在肿瘤基础研究中，近年来对作为“土壤”的肿瘤微环境的探索逐渐增多。作为肿瘤微环境的主要成分，癌相关成纤维细胞（cancer-associated fibroblasts, CAFs）在肿瘤发生、进展及预后中的作用被逐步阐明。而CAFs表型形成机制的阐明将有助于更深刻的理解其在肿瘤中的作用。CAFs主要有四种起源：上皮细胞、内皮细胞、间质干细胞和局部的间质细胞。研究发现，不同起源的CAFs具有大致相同的表型特征，而CAFs表型转换的机制却不甚清楚。目前的研究主要集中在：①CAFs基因的改变; ②CAFs表观遗传学改变。本文将在阐述CAFs起源多样性和表型特征一致性的基础上，系统总结CAFs表型转换的表观遗传学机制的现状和最新进展。

## Abstract (en)

In the field of human cancer research, even though the vast majority attentions were paid to tumor cells as "the seeds", the roles of tumor microenvironments as "the soil" are gradually explored in recent years. As a dominant compartment of tumor microenvironments, cancer-associated fibroblasts (CAFs) were discovered to correlated with tumorigenesis, tumor progression and prognosis. And the exploration of the mechanisms of CAF phenotype transformation would conducive to the further understand of the CAFs function in human cancers. As we known that CAFs have four main origins, including epithelial cells, endothelial cells, mesenchymal stem cells (MSCs) and local mesenchymal cells. However, researchers found that all these origins finally conduct similiar phenotypes from intrinsic to extrinsic ones. Thus, what and how a mechanism can conduct the phenotype transformation of CAFs with different origins? Two viewpoints are proposed to try to answer the quetsion, involving genetic alterations and epigenetic modifications. This review will systematically summarize the advancement of mechanisms of CAF phenotype transformations in the aspect of genentic and epigenetic modifications.

1889年，Stephen Paget提出肿瘤转移的“种子和土壤”学说^[1]^。至今，作为“种子（seeds）”的肿瘤细胞在肿瘤演进中的关键作用已得到深入的探讨。而肿瘤微环境（土壤）作为另一重要组分在肿瘤发生、进展及预后中的作用，只是在最近才被逐渐重视。肿瘤微环境主要包含细胞外基质（extracellular cell matrix, ECM）和各种细胞（如炎性细胞、免疫细胞等）。作为肿瘤微环境中主要的细胞种类，癌相关成纤维细胞（cancer-associated fibroblasts, CAFs）通过与肿瘤细胞的“cross-talk”影响肿瘤的进展和预后^[2]^。Martinez-Outschoorn等^[3]^提出“肿瘤代谢的间质自噬模型（autophagic tumor stroma model of cancer metabolism）”，发现肿瘤间质（主要是CAFs）在不依赖血管增生的情况下，通过自噬为肿瘤细胞的生长、侵袭和转移提供“燃料（battery）”。CAFs分泌的多种细胞因子与肿瘤预后相关，包括各种生长因子（growth factors）^[4]^、促炎性因子（pro-inflammatory factors）^[5]^、基质金属蛋白酶家族（matrix metalloproteases）^[6]^等。然而，若要更加深入探索CAFs在肿瘤中的作用，我们必须着眼于CAFs的表型形成机制。本文将从以下三方面进行系统的论述：CAFs表型一致性与起源多样性; CAFs表型转换的遗传学基础（genetic alterations）; CAFs表型转换的表观遗传学改变（epigenetic modifications）。

## 1 CAFs表型一致性与起源多样性

作为一种特殊的肿瘤相关细胞，CAFs在病理学特征上与活化的成纤维细胞（activated fibroblasts）相似。研究发现，在不同组织来源的肿瘤中，CAFs拥有许多相同或相似的表型特征。因此，Shalom等^[7]^最近提出一个新定义——“CAFs状态（CAFs state）”，认为“CAFs”应该是肿瘤间质中一群保持动态活性的促进肿瘤进展的成纤维样细胞。

在内在特征方面，CAFs与正常成纤维细胞有显著差异。在细胞形态上，拥有更大纺锤形细胞形态; 在细胞标志物上，拥有一些与肌成纤维细胞、肌上皮细胞相同的标志物，如α-SMA。此外，CAFs的增殖、分裂能力较正常成纤维细胞显著增强，且能分泌多种细胞因子和细胞外基质。然而，与肿瘤细胞相关的外在特征被认为是鉴别CAFs的更为重要的因素。首先，CAFs分泌多种因子促进肿瘤细胞的生长、浸润，包括血小板衍生生长因子受体（platelet-derived growth factor, PDGFR）^[8]^、白介素-1β（interleukin 1β, IL-1β）^[9]^等。其次，CAFs通过分泌赖氨酸氧化酶（Lysyl oxidase, LOX），对细胞外基质进行重构进而促进肿瘤细胞的转移侵袭^[10]^。此外，Mani等^[11]^发现，CAFs可介导肿瘤细胞的上皮间质转化（epithelial-mesenchymal transition, EMT），进而促进肿瘤的转移侵袭。研究^[12]^还发现CAFs与肿瘤耐药性相关。

虽然不同肿瘤的CAFs间有表型共性，但是研究发现CAFs却有不同的细胞起源，主要包括上皮细胞（epithelial cells）、内皮细胞（endothelial cells）、间质干细胞（mesenchymal stem cells, MSCs）和局部的间质细胞（local mesenchymal cells）。EMT使得上皮细胞成为CAFs的一种可能的起源。研究^[13]^发现，老鼠皮肤鳞癌细胞可通过激活Ras/TGF-β信号途径而获得间质细胞的表型特征。内皮细胞可经内皮间质转化（endothelial to mesenchymal transition, EndMT）过程而获得间质表型，进而形成CAFs相关表型。Zeisberg等^[14]^即发现，在B16F10黑色素瘤模型和Rip-Tag2自发性胰腺癌模型中均能频繁发生内皮间质转化过程。间质干细胞是一种被认为在肿瘤发生、进展中发挥重要作用的细胞，亦被认为可能是CAFs的一个起源。Worthley等^[15]^在曾接受骨髓移植患者的胃癌中，发现绝大部分CAFs起源于移植的骨髓细胞。此外，来自肿瘤周围的局部间质细胞也可能是CAFs重要起源。

CAFs起源的多种可能，向我们揭示了不同起源之间在表型转换过程中存在着某些重要且共通的机制，而且这些机制可能与其周围的肿瘤细胞存在着密切的关系，以至于不同肿瘤中CAFs拥有许多共通的表型特性。当然，这过程中我们也需要重视他们之间的个性差异。目前的研究主要集中在CAFs基因改变和表观遗传学改变等两方面。

## 2 CAFs表型转换的遗传学基础

众所周知，由一个受精卵分裂增殖而形成的生物有机体的不同细胞间共享一套遗传信息（DNA），然而又表现出不同的表型特征和生理功能。研究发现，决定一个细胞表型特征变化的因素除了DNA的改变之外，表观遗传学的改变亦在其中发挥关键性的作用。学界也试图从此两方面对CAFs表型转换机制进行探讨。

研究首先是从基因层面进行，学者们对CAFs可能发生基因序列改变的情况进行了相关研究。Kurose等^[16]^发现，在乳腺癌的CAFs中存在*PTEN*和*TP53*体细胞突变（somatic mutation）。更多的研究^[17-20]^却提示CAFs存在杂合性丢失（loss of heterozygosity, LOH）、微卫星不稳定性（microsatellite instability, MSI）和等位基因失衡（allelic imbalance, AI）等。Fukino等^[18]^对134例散发浸润性乳腺癌的上皮和间质成分进行全基因组LOH检测，发现至少有57个LOH位点在上皮（*n*=19）和间质（*n*=38）中均存在，肿瘤间质LOH的多样性可能与宿主间质对侵袭性腺癌细胞的反应的个体差异有关，Matsumoto等^[19]^对40例散发性结直肠癌的上皮和间质成分进行MSI研究，发现间质和上皮的MSI阳性率分别为41%和34%。进一步研究发现，间质MSI与上皮P53蛋白高表达呈负相关（*P*=0.024, 75）。此外，Tanwar等^[20]^发现，通过定向敲除小鼠子宫间质细胞的结肠腺瘤性息肉病基因（adenomatous polyposis coli, APC）后，小鼠子宫内膜腺体出现异型增生，进而发展出子宫内膜原位腺癌及侵袭性腺癌。他们还发现肿瘤细胞周围的间质细胞表达α-SMA，具有肌成纤维细胞样的表型特征。进而，研究者们提出了一个大胆的假说，认为CAFs和肿瘤细胞可能相互依存、共同演进^[21]^。然而，另一些研究发现，肿瘤间质中的CAFs并不存在遗传信息改变。例如，Karakas等^[22]^分别对81例原发侵袭性乳腺癌和25例乳腺癌CAFs标本的*PIK3CA*基因的3个热点突变进行检测，发现在CAFs中没有体细胞突变。同样的，Corver等^[23]^通过单核苷酸多态性芯片技术对57例宫颈癌CAFs的LOH、DNA异倍体等进行研究，发现其DNA为二倍体，没有体细胞突变的任何证据。这种基于实验结果不同而产生的矛盾，将我们的目光拉回到那个假说在理论层面更为深刻的矛盾上去。即，为何CAFs会屈尊以支持肿瘤细胞的生长，而自身却不进展为肉瘤细胞; 或是共同演进而出现一个实体肿瘤中存在两种肿瘤细胞（癌和肉瘤）？虽然癌肉瘤偶有病案报告，但是并不足以支持该假说。近年来，研究者们将目光转向另一方面——CAFs的表观遗传学改变。

虽然表观遗传现象早在1940年代即被发现，但是直到近期才被逐渐阐明。目前认为，表观遗传学是研究可遗传的、没有基因序列改变的基因功能的改变（或是细胞表型转换）机制的学科^[24]^。当DNA的奥秘被逐一解开之后，我们曾一度认为细胞若要改变其表型特征，相对应的DNA序列必定要发生改变。随着实验技术的发展，人们逐渐发现，表观遗传学的改变也会导致细胞的父系表型发生变化，并可通过有丝分裂或减数分裂遗传给子代细胞^[25]^。表观遗传学机制存在于转录、转录后mRNA修饰、翻译等水平，通过对基因表达的不同水平进行调控进而影响细胞表型。根据不同的基因表达水平，表观遗传学机制主要包括DNA甲基化、基因印记、组蛋白修饰、RNA干扰等。研究发现表观遗传学改变在肿瘤、自身免疫性疾病、遗传性疾病等中发挥重要作用。

虽然肿瘤的表观遗传学研究已很深入，但是目前对CAFs表型转换的表观遗传学机制研究仍偏少。表观遗传学的关键是不改变DNA核苷酸序列和可遗传性^[26]^。基于这种特性，表观遗传学为我们提供了一种观点，使我们能够初步理解CAFs的起源与表型特征之间的矛盾。因此，我们将总结目前关于CAFs表型转换的表观遗传学机制的研究进展，为以后的研究提供参考。

## 3 CAFs表型转换的表观遗传学改变

我们将从DNA甲基化（DNA methylation）、组蛋白修饰（histone modification）和microRNA干扰（miRNA interference）三方面对CAFs表型转换与表观遗传学改变的研究进展进行全面论述。

### 3.1 DNA甲基化

DNA甲基化尽管在不同区域都可发生（如CpNpG甲基化），但是绝大部分都出现在5’-C端CpG岛的胞嘧啶（cytosine, C）。对下游靶基因的转录可产生促进或抑制的两种效应，多数情况下DNA甲基化抑制靶基因的表达。其机制主要为通过募集甲基化CpG结合蛋白（methyl-CpG-binding domain proteins）或直接抑制DNA结合蛋白（DNA binding proteins）的募集而抑制基因的转录过程^[27]^。

研究发现，肿瘤细胞通过旁分泌IL-6对周边的CAFs进行表观遗传学调控，使*TGFBR2*基因表达下调。在前列腺癌中，研究进一步发现CAFs的*GSTP1*和*TGFBR2*基因的DNA甲基化将导致其DNA损伤和细胞的脆弱性增加^[28]^。众所周知，CAFs的DNA损伤和细胞脆性将促进肿瘤细胞的生长。因此，肿瘤细胞可通过表观遗传学途径增加CAFs的DNA损伤和细胞脆性并促进自身的生长。尽管具体机制尚不清楚，有证据显示其可能与TGF-β信号途径受抑制以及DNA甲基化酶1（DNMT1）的稳定性相关，但是在这方面仍需要更多深入的研究。

此外，宫颈癌CAFs的*OPCML*基因和前列腺癌CAFs的*HIC1*、*N33*和*RARB2*等抑癌基因也存在DNA甲基化。Fiegl等^[29]^用激光捕获的显微切割方法对143例乳腺癌间质进行DNA甲基化状态研究，发现HER-2/neu阳性患者的5个基因（包括*MYOD1*、HSD17*B4*、*CDH1*3、*PGR*和*BRCA1*）的DNA甲基化显著高于（*P*=0.011）HER-2/neu阴性患者。其中，MYOD1和CDH13与肿瘤细胞的转移、侵袭相关，而*PGR*基因甲基化则导致雌激素受体丢失。在对食管癌的肿瘤细胞和CAFs的*COX-2*基因（PTGS2）DNA甲基化进行对比研究后，Dawsey等^[30]^发现存在DNA甲基化的CAFs均靠近同样存在*COX-2*基因DNA甲基化的肿瘤细胞。他们猜测，肿瘤细胞可能通过相互作用影响周围的间质细胞，使之亦出现DNA甲基化并诱导CAFs表型转换，进而促进肿瘤发生。尽管在多种类型肿瘤CAFs中均发现DNA甲基化的存在，但是仍缺乏有力的证据来对其与CAFs表型变化和肿瘤发生、进展之间关系进行系统说明。

### 3.2 MicroRNA干扰

MicroRNA是一条由RNA聚合酶Ⅱ（RNA polymerase Ⅱ）转录并最终在胞质成熟的约有22个核苷酸的RNA。它可以通过特异性结合mRNA而干扰其翻译过程，进而诱导基因表达沉默。根据miRNA与mRNA之间结合的紧密程度不同，其干扰机制也有所差异。当miRNA与mRNA完全紧密结合时，导致mRNA稳定性下降并最终被降解; 反之，则仅是翻译过程被抑制。在CAFs中，多种miRNAs与CAFs表型转换相关，并促进肿瘤的发生、侵袭和转移。

Mitra等^[31]^发现，相较于正常成纤维细胞，在卵巢癌CAFs中miRNA-31和miRNA-214表达下调，而miRNA-155则表达上升。通过在正常成纤维细胞中，用转染miRNA和miRNA抑制等方法模拟CAFs中miRNAs表达失调过程时，他们发现正常成纤维细胞表现出许多与CAFs相同的表型特征，包括自身克隆形成率的上调、促肿瘤细胞生长、转移和侵袭、以及多种基因的表达上调（主要是细胞因子相关的基因）。更重要的是，在CAFs中逆向模拟miRNAs表达谱时，CAFs的表型特征向着正常成纤维细胞转变。该研究提示，miRNA在CAFs表型形成过程中可能发挥着重要作用，并且，与卵巢癌细胞共培养后，正常成纤维细胞内的miRNAs表达升高。研究^[32]^也发现miRNA-21能通过TGF-β1介导影响CAFs的表型转换。在肿瘤代谢方面，Pavlides等^[33]^发现，miRNA-34c/-31参与CAFs的“反沃伯格效应（Reverse Warburg Effect）”，使CAFs向肿瘤细胞提供增殖所必须的各种营养物质。研究CAFs与肿瘤相关炎症时，Olga等^[34]^发现，CAFs中miRNA-146a表达上调能够通过激活NF-κB信号途径进而促进肿瘤相关炎症反应。此外，CAFs中的许多miRNAs表达异常均能促进肿瘤发生、进展及转移，包括miRNA-148a^[35]^、miRNA-214/-221/-222^[36]^、miRNA-21^[37]^、miRNA-15/-16^[38]^、miRNA-320^[39]^等。尽管研究发现CAFs中多种miRNAs表达改变与表型转换、促肿瘤生长侵袭等相关，但是这些miRNAs的表达改变是否与肿瘤细胞相关，肿瘤细胞通过何种机制调节CAFs中miRNAs的表达等，仍需进一步的探索。

### 3.3 组蛋白修饰

组蛋白修饰在转录调节、DNA修复、DNA复制、染色体浓缩等过程中发挥着重要作用。研究^[40]^发现，组蛋白的修饰主要发生在N-末端，主要有乙酰化（acetylation）、甲基化（methylation）、磷酸化（phosphorylation）、泛素化（ubiquitination）等形式。并且，Lachner等^[41]^发现不同组氨酸部位的赖氨酸甲基化作用亦各不相同。Tyan等^[42]^发现，在CAFs中EZH2（enhancer of zeste homologue 2）的丢失将导致组蛋白H3K27去甲基化（demethylation），进而使ADAMTS1表达上调。CAFs中ADAMTS1表达上调与肿瘤的转移、侵袭相关。此外，Zheng等^[43]^发现，EZH2促进组蛋白H3K27甲基化，进而通过启动子的DNA甲基化使*p16INK4a*基因表达沉默。而EZH2到p16INK4a调控通路与MSCs的衰老和恶变等表型转换相关。目前关于CAFs组蛋白修饰的表观遗传学机制研究仍较少，需要更多深入的探讨以进一步阐明组蛋白修饰在CAFs表型转换中的作用。此外，组蛋白修饰与DNA甲基化可能存在相互作用，进而调节靶基因的表达以改变CAFs表型。

## 4 问题与展望

在CAFs表型转换的表观遗传学研究主要集中在DNA甲基化、miRNAs和组蛋白修饰等三方面。虽然目前的研究提示CAFs表观遗传学改变在其表型转换及与肿瘤生长、侵袭等方面可能发挥着重要的作用，但是这些表观遗传学改变与CAFs表型转换之间是何种关系，是否与肿瘤细胞存在必然的联系，CAFs的表观遗传学改变对肿瘤细胞的生长、侵袭是否产生明确的影响等问题仍然有待更深入的研究。此外，CAFs表观遗传学机制多数只在一种肿瘤中发现或验证，由于不同类型的肿瘤之间既有共性也有个性，对在某一类肿瘤中可能有效的机制，因此还需要更进一步的研究以探索相关机制的普适性。

综上所述，CAFs表型转换的表观遗传学研究仍处于起步阶段，多方面的深入研究等待我们跟进，包括不同肿瘤之间是否存在相同或相似的表观遗传学机制（DNA甲基化、组蛋白修饰、RNA干扰等）; 肿瘤细胞是通过何种具体的表观遗传学机制影响CAFs表型转换; 是否还存在其他的表观遗传学机制; CAFs的表观遗传学改变与基因序列的改变是否有关等等。通过阐明CAFs表型转换的表观遗传学机制，有助于我们深入理解CAFs在肿瘤发生、进展及预后中的作用。
